# Development of a soft X-ray angle-resolved photoemission system applicable to 100 µm crystals

**DOI:** 10.1107/S0909049511034418

**Published:** 2011-09-21

**Authors:** Takayuki Muro, Yukako Kato, Tomohiro Matsushita, Toyohiko Kinoshita, Yoshio Watanabe, Hiroyuki Okazaki, Takayoshi Yokoya, Akira Sekiyama, Shigemasa Suga

**Affiliations:** a Japan Synchrotron Radiation Research Institute (JASRI), 1-1-1 Kouto, Sayo, Hyogo 679-5198, Japan; b The Graduate School of Natural Science and Technology, Okayama University, 3-1-1 Tsushima-naka, Okayama 700-8530, Japan; c Graduate School of Engineering Science, Osaka University, 1-3 Machikaneyama, Toyonaka, Osaka 560-8531, Japan

**Keywords:** angle-resolved photoemission spectroscopy (ARPES), soft X-ray, small crystal, microcleaving, micropositioning

## Abstract

A system for angle-resolved photoemission spectroscopy (ARPES) of small single crystals with sizes down to 100 µm has been developed. Soft X-ray synchrotron radiation with a spot size of ∼40 µm × 65 µm at the sample position is used for the excitation. Using this system an ARPES measurement has been performed on a Si crystal of size 120 µm × 100 µm × 80 µm. The crystal was properly oriented on a sample stage by measuring the Laue spots. The crystal was cleaved *in situ* with a microcleaver at 100 K. The cleaved surface was adjusted to the beam spot using an optical microscope. Consequently, clear band dispersions along the Γ–*X* direction reflecting the bulk electronic states were observed with a photon energy of 879 eV.

## Abstract 2

A soft X-ray angle-resolved photoemission system applicable to 100 µm crystals has been developed.

## 1. Introduction

Angle-resolved photoemission spectroscopy (ARPES) is a powerful technique for observing electronic band structures and Fermi surfaces of solids. ARPES measurements require the use of single crystals. Typically, crystal sizes of more than a few millimeters are preferred to facilitate sample treatment and/or overcome the limitation of the photon beam size. However, it is often difficult to obtain such single crystals, especially in the case of newly synthesized compounds. The crystal sizes of such materials are mostly as small as 100 µm or less. In order to reveal their electronic structures, an ARPES system applicable to such small crystals is desired.

For ARPES measurements of small crystals the excitation light should be focused into the smallest possible size. In the case of third-generation synchrotron light sources, ultraviolet light and soft X-rays can easily be focused into spots with sizes of less than 100 µm using grazing-incidence focusing mirrors without significant loss of photon flux. This feature enables the use of ARPES for crystals down to 100 µm. However, scaling down the crystal size and light spot leads to some technical difficulties. For example, one must optimize the 100 µm sample position in a vacuum chamber relative to the small light spot. One must also finely tune the sample position following repeated changes of the angle of the sample during the ARPES measurements. An *in situ* cleaned surface has to be obtained even for such a small sample. Still, the crystal orientation must be ensured to provide band dispersions along certain symmetry axes in *k*-space.

To overcome these difficulties we recently reported such elemental techniques as sample-positioning methods with magnifying video cameras (Muro *et al.*, 2009*a* ▶,*b* ▶) and an ultrahigh-vacuum compatible microcleaver for obtaining *in situ* cleaned surfaces of small crystals (Muro *et al.*, 2010 ▶). By combining these techniques with focused soft X-rays of the synchrotron facility SPring-8, we have developed an ARPES system for measuring 100 µm crystals. Soft X-ray ARPES can probe electronic structures with increased bulk sensitivity compared with the conventional ARPES in the photon energy region 15–200 eV and has played important roles in studying correlated materials and superconductors (Sekiyama *et al.*, 2004 ▶; Yokoya *et al.*, 2005 ▶; Yano *et al.*, 2007 ▶). In this paper we report the details of the developed system and the results of a demonstration experiment on a Si crystal of size 120 µm × 100 µm × 80 µm.

## 2. Experimental system

**Figure 1:**
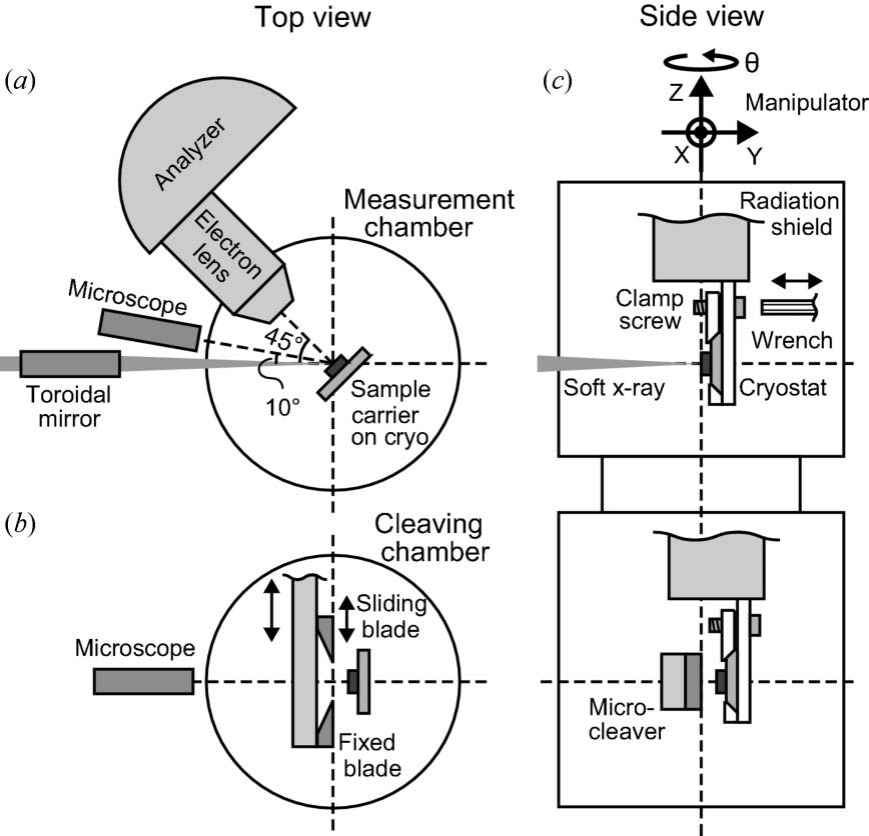
Schematic layout of the ARPES system: (*a*) top view at the measurement level; (*b*) top view at the cleaving level; and (*c*) side view of the measurement and cleaving chambers.

The system has been developed at the soft X-ray helical undulator beamline BL25SU (Saitoh *et al.*, 1998 ▶, 2000 ▶) at SPring-8. The monochromator covers the photon energy range from 0.12 to 2 keV with a typical resolving power of about 10000. Fig. 1 ▶ shows a schematic layout of the station. Soft X-rays converge on the sample position using a toroidal mirror placed 1.25 m upstream, as shown in Fig. 1(*a*) ▶. The measured vertical and horizontal FWHMs of the beam spot at the sample position (normal to the optical axis) are 40 µm and 65 µm, respectively. A sample mounted on a sample carrier is installed in a vacuum in the load lock chamber (not shown in the figure) and then transferred on a cryostat in the measurement chamber with a base pressure of ∼2 × 10^−8^ Pa. The holder of the sample carrier, shown in the upper part of Fig. 1(*c*) ▶, has a clamp screw, which can be tightened using a retractable wobble stick with a hexagon wrench to obtain a good thermal contact between the carrier and the cryostat. We use a closed-cycle helium refrigerator instead of a flow-type cryostat in order to save on the consumption of helium. The lowest temperature of the cryostat at the sample holder position is about 8 K. As indicated in Fig. 1(*c*) ▶, the sample manipulator has an *XYZ* linear stage and a rotary θ stage driven by stepping motors. The *X*, *Y* and *Z* axes have linear encoders with resolutions of 0.5 µm, which are sufficient for positioning 100 µm crystals. The θ stage also has a rotary encoder with a resolution of 0.01°.

**Figure 2:**
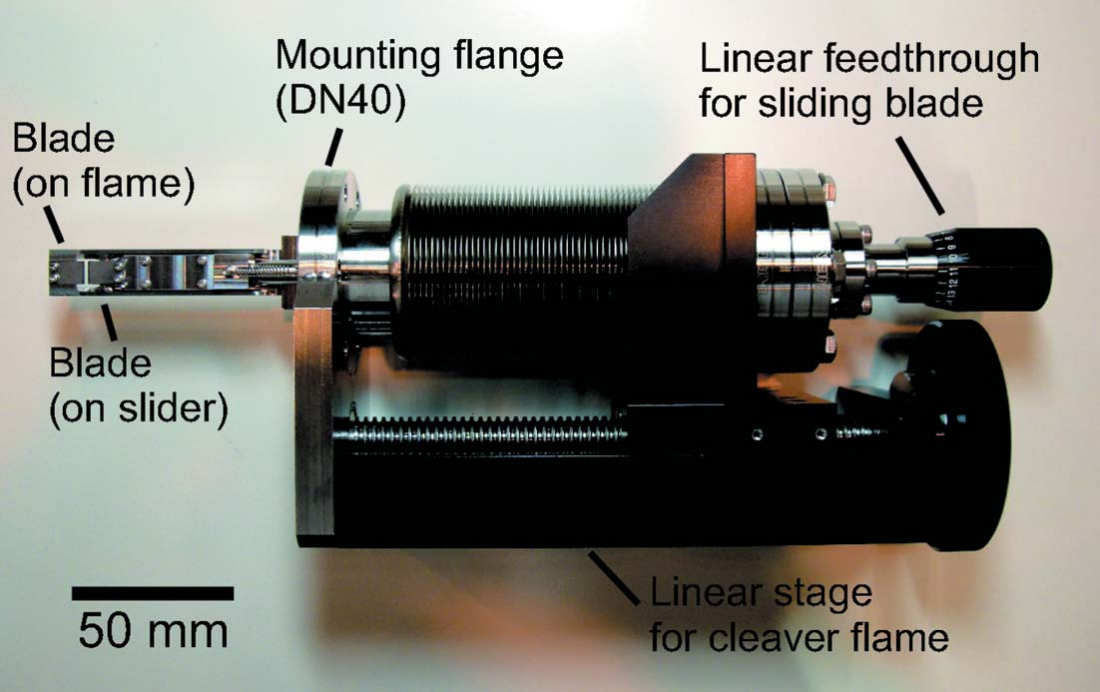
Photograph of the microcleaver. For details of the parts around the blades, see Fig. 2 ▶ of Muro *et al.* (2010 ▶).

**Figure 3:**
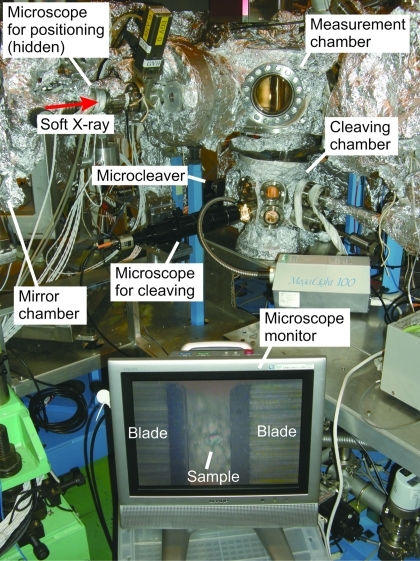
Photograph of the ARPES system including the microcleaving system; a Si crystal with a size of about 100 µm and the cleaver blades are seen in the microscope monitor.

Before the ARPES measurements, the sample is lowered to the cleaving level, which is shown in the lower part of Fig. 1(*c*) ▶, and cleaved by a microcleaver. Fig. 2 ▶ shows a photograph of the cleaver dismounted from the chamber. This cleaver is manually controlled, whereas the previous one (Muro *et al.*, 2010 ▶), now installed at another beamline, employed stepping motors for a target size of a few tens of micrometers. For the present purpose of cleaving ∼100 µm crystals, a manually controlled cleaver is also applicable. The mechanism is basically the same as the previous one except for the motion control (for details, see Muro *et al.*, 2010 ▶). The cleaver has a pair of sharp blades, as shown in Fig. 1(*b*) ▶. One blade is fixed on the cleaver frame. The other blade slides on the frame toward the fixed one with perpendicular positional deviations of less than ±5.5 µm, which are sufficiently small to nip a ∼100 µm crystal. The cleaver has two manual linear feedthroughs, one moves the cleaver frame and the other moves the sliding blade. When we cleave a sample (Fig. 1*b* ▶), first, the sample is horizontally retracted from the center of the chamber and the blades with a gap of several millimeters are placed there. Then, the sample is adjusted back into the gap and manipulated to touch the tip of the fixed blade using the *XY* sample stage. Finally, the sliding blade cuts into the sample and cleaves it. Since it is almost impossible to carry out these operations by the naked eye, the sample and the blades are monitored using an optical microscope (Infinity Photo-Optical, K2/S) mounted on a viewport, as shown in Fig. 3 ▶. The working distance of the microscope is 160 mm and the total magnification is about 310 when a camera with a 1/3-inch CCD and a 13-inch monitor are used. In our system, samples can be cleaved at low temperatures, which is helpful to obtain better-cleaved surfaces.

After cleaving, the sample is lifted up to the measurement level. An optical microscope is again used to adjust the cleaved area to the measurement point as shown in Fig. 1(*a*) ▶. The working distance is 410 mm and the total magnification is 280 with a 1/3-inch CCD and a 13-inch monitor. Since the details of the positioning method have already been reported (Muro *et al.*, 2009*a* ▶), we only briefly describe the method here. The microscope horizontally monitors the measurement point, where the soft X-ray beam and the electron lens axis intersect, at an angle of 10° from the beam. The measurement point is marked on the microscope monitor in advance using a phosphor plate. The cleaved surface is adjusted to this mark on the monitor by the *XYZ* motion. At this stage, however, the sample surface is not correctly placed at the measurement point because of the focal depth of the microscope of about 1 mm. This ambiguity can be fixed by scanning the sample along the microscope axis keeping the sample image at the mark, and finding the position where the analyzer detects the maximum photoelectron counts. The horizontal region from which the analyzer can detect photoelectrons is much narrower than the focal depth of the microscope. For example, the region is about 40 µm on the sample surface set normal to the electron lens axis, as in Fig. 1(*a*) ▶, when using an imaging lens mode with a magnification of 5 and a slit width of 0.2 mm. The above-mentioned procedure corresponds to determining the optimal sample position by observing it from the two directions defined by the microscope and the electron lens axes.

ARPES spectra are measured with a hemispherical electron analyzer (VG Scienta AB, SES200). This analyzer can detect angle-dispersed photoelectrons along the longitudinal direction of the entrance slit as a function of the photoelectron kinetic energy. In our configuration the longitudinal direction of the slit is set vertical. Then, the angle-resolved spectra are measured with an angular resolution of 0.2° within ±6° along the slit. By using this angle-dispersive detection of the analyzer and the θ rotation of the sample manipulator, two-dimensional intensity maps of the angle-resolved photoelectrons can be measured. The use of high excitation energies of soft X-rays enables us to observe band dispersions in a wider range in *k*-space than the use of ultraviolet for a given angular acceptance. In most cases the angular acceptance of ±6° is sufficient to cover the full first Brillouin zone. Hence, Fermi surfaces covering the first Brillouin zone can be observed by this intensity mapping (Sekiyama *et al.*, 2004 ▶; Yokoya *et al.*, 2005 ▶; Yano *et al.*, 2007 ▶).

## 3. Performance

### 3.1. Sample preparation

**Figure 4:**
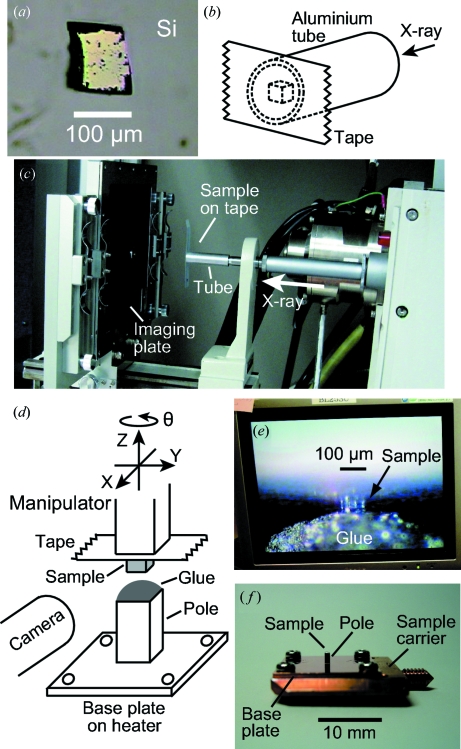
(*a*) A 120 µm × 100 µm × 80 µm Si crystal for the ARPES measurement. (*b*) Schematic view of the double-sided tape and aluminium tube, which were used for the Laue measurement. (*c*) Laue camera system, where the aluminium tube was put at the exit of the X-ray source. The sample attached on the tape was located in the path of the X-ray beam. (*d*) Schematic drawing of the system for mounting the sample onto the electroconductive glue applied on the pole of cross section 1 mm × 1 mm. The sample position relative to the glue is horizontally monitored by two cameras (one is not shown in the figure) and adjusted by the *XYZ* stage. (*e*) One of the camera monitors, which showed the sample, the lower half of which was embedded in the electroconductive glue. (*f*) Sample carrier on which the base plate was screwed.

Using this system we have carried out an ARPES measurement for a Si crystal of size 120 µm × 100 µm × 80 µm shown in Fig. 4(*a*) ▶. In order to observe the band dispersions of the crystal along a high-symmetry direction in *k*-space, we first checked the crystal orientation with a laboratory-scale Laue camera with a Cu rotating-anode X-ray generator. Because the sample-positioning stage of the Laue camera was not suitable for adjusting a 100 µm crystal, we mounted the crystal directly on the exit aperture of the X-ray source using double-sided adhesive tape and an aluminium tube, as shown in Figs. 4(*b*) and 4(*c*) ▶, where the sample surface seen in Fig. 4(*a*) ▶ was attached to the tape. The X-ray passed through the sample with a thickness of 80 µm and weak but sharp Laue spots were observed, confirming the (111) surface orientation. Then, we tried to mount this sample on the sample carrier with proper orientation. First, the sample was mounted on a Cu pole standing on a Cu base plate using an *XYZ*θ manipulator as shown in Fig. 4(*d*) ▶, where the tape and the base plate were set parallel. The purpose of using the pole was to facilitate cleaving. The base plate was placed on a heater. Since the in-plane orientation of the (111) surface relative to the tape was known by the Laue spots, we were able to orient the sample relative to the base plate by adjusting the in-plane angle between the tape and the base plate. After adjusting the angle, the lower half of the sample was embedded in electroconductive glue applied on the pole by the *XYZ* motion, as seen in Fig. 4(*e*) ▶, and the glue was coagulated by heating the base plate. Then, the manipulator was lifted and the sample, unstuck from the tape, was left on the glue. Finally, the base plate was screwed on the carrier as shown in Fig. 4(*f*) ▶. In order to observe the band dispersions along the Γ–*X* direction, the [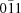] direction was oriented on the carrier along the angle-dispersive direction of the analyzer when it was mounted on the sample holder.

**Figure 5:**
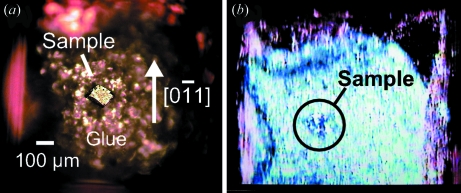
(*a*) Si crystal mounted on the electroconductive glue observed by an optical microscope before it was installed in the vacuum. The crystal orientation is also indicated. (*b*) Si crystal in the cleaving chamber, observed through a viewport after cleaving.

After the sample was transferred into the vacuum chamber, it was cooled down to 100 K in order to obtain a high-quality cleaved surface. Then the sample was cleaved with the microcleaver following the procedure described in §2. In our system the sample holder is electrically insulated from the ground and wired to an electrical feedthrough. Therefore, we were able to notice the mechanical contact between the sample and the grounded blades by monitoring their electrical contact. This was helpful for positioning of the sample relative to the blades, especially in adjusting it along the direction of the focal depth of the microscope, *i.e.* the *Y* direction in Fig. 1(*c*) ▶. Fig. 5(*b*) ▶ shows the cleaved crystal surface observed by the microscope. The specular surface implies successful cleavage along the (111) cleavage plane of Si. For comparison, Fig. 5(*a*) ▶ shows the sample observed with an optical microscope and the crystal orientation before it was installed in the vacuum. The thickness of the portion of the sample outside the glue was about 40 µm before cleaving as shown in Fig. 4(*e*) ▶. In order to nip this 40 µm thickness, the relative positions of the blades along the direction of the sample thickness, *i.e.* the *Y* direction in Fig. 1(*c*) ▶, were finely tuned before the cleaver was installed on the cleaving chamber.

### 3.2. Results and discussion

**Figure 6:**
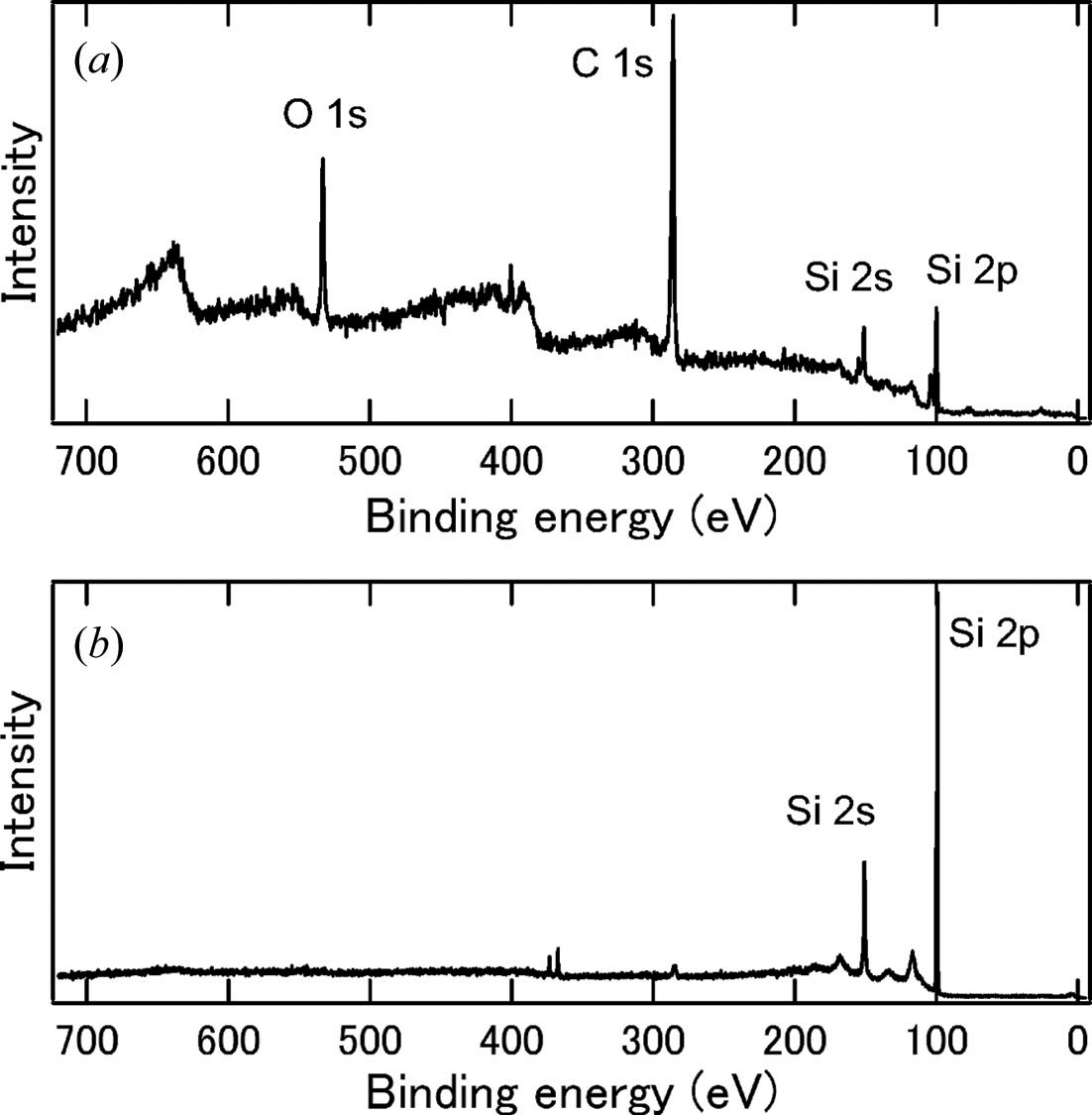
Angle-integrated photoemission spectra measured (*a*) before cleaving and (*b*) after cleaving. For details of the doublet peak structure at the binding energy of 370 eV, see text.

Fig. 6 ▶ shows the angle-integrated photoemission spectra including the Si 2*p*, Si 2*s*, C 1*s* and O 1*s* core levels measured for the Si crystal before and after cleaving. A photon energy of 898 eV was used. The crystal was adjusted to the measurement position by the positioning method described in §2. In the spectrum before cleaving, the C 1*s* and O 1*s* structures overwhelm the Si 2*p* and 2*s* structures, but they almost disappear after cleaving. This means that cleaving successfully produced a clean surface.

**Figure 7:**
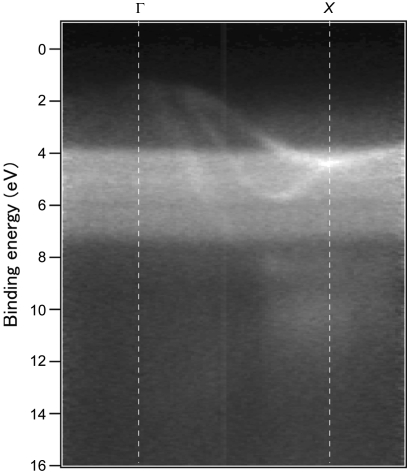
Intensity map of ARPES spectra measured for the cleaved Si in Fig. 5(*b*) ▶ along the [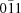] direction. For details of the angle-independent spectral weight in the binding-energy range from 4 to 8 eV, see text.

Then, we measured the ARPES spectra along the [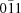] direction. The sample temperature was maintained at 100 K. Although sample cooling was not necessary to observe the band dispersions of Si, we tried to check the capability of measuring a 100 µm crystal under temperature-controlled conditions. The observed spectra show clear band dispersions along the Γ–*X* direction as seen in Fig. 7 ▶. A photon energy of 879 eV was used. The total energy resolution was set at 260 meV. The sample position was monitored by the microscope during the measurement. There were some vibrations caused by the motion of the He refrigerator at ∼2.5 Hz, which are discussed later. However, no long-period positional drifts were observed, even though the sample temperature was proportional-integral-derivative (PID) controlled with a ceramic heater. The observed dispersions correspond well to the ones previously observed for a millimeter-sized Si crystal along the Γ–*X* direction (Muro *et al.*, 2009*a* ▶), which implies that the present 100 µm crystal was well oriented. The surface states expected for the cleaved Si(111) surface, which are distributed mainly in the binding energy range from 0 to 1 eV (Himpsel *et al.*, 1981 ▶), are not seen in Fig. 7 ▶, reflecting the bulk sensitivity of the high-energy excitation. The angle-resolved spectra in Fig. 7 ▶ were measured using the angle-dispersive detection of the analyzer without changing the relative angle between the sample and the analyzer.

In Fig. 7 ▶, however, a background-like non-dispersive structure is also seen in the binding energy range from 4 to 8 eV. This structure is attributed to the Ag 4*d* valence band signals coming from the electroconductive glue surrounding the crystal. This appears to primarily result from the light spreading out of the Si crystal. Since the vertical FWHM of the light spot was 40 µm and less than half of the crystal size, the contribution of the vertical spread is expected to be negligible. Conversely, the horizontal FWHM was 65 µm when θ = 0°, *i.e.* the beam normally impinged on the sample surface and should be expanded to 92 µm when θ = 45° for the ARPES measurement. If we assume a Gaussian-like profile for the horizontal photon intensity distribution, about 20% of the total intensity is spread out of the 100 µm crystal. Furthermore, the cross section of the Ag 4*d* states is about 90 (23) times larger than that of the Si 3*p* (3*s*) states (Yeh & Lindau, 1985 ▶). This must pronounce the Ag 4*d* signals relative to the Si valence signals. Ag 3*d* core signals are also seen in Fig. 6(*b*) ▶ at around 370 eV, but they are less pronounced because the spectrum was measured at θ = 0°.

In order to reduce the horizontal spot size, we are planning further tuning of the focusing optics. The ideal horizontal size expected from the source size and the demagnification is about 25 µm. It is usually difficult to achieve the best focusing for both the horizontal and vertical spot sizes with a single toroidal mirror. In our beamline, however, the horizontal size can be tuned not only by the postfocusing toroidal mirror but also by a prefocusing bendable cylinder (Saitoh *et al.*, 1998 ▶). We expect that the horizontal size will be reduced to less than 40 µm by optimizing the bend radius without changing the vertical size, which is small enough for 100 µm crystals.

**Figure 8:**
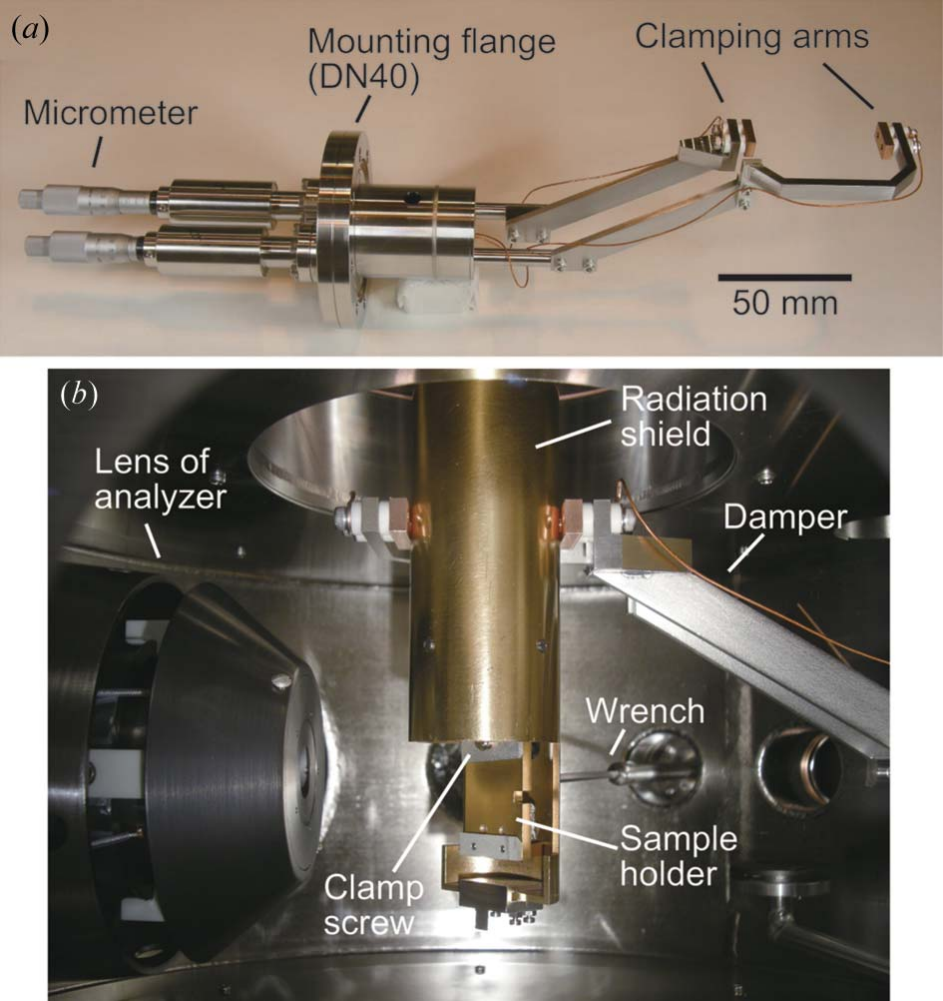
(*a*) Vibration damper for the cryostat. (*b*) Photograph of the inside of the measurement chamber, where the radiation shield of the cryostat is clamped by the damper.

The periodic motion of the closed-cycle He refrigerator could also be a reason for the enhancement of the photoelectron signals from the surrounding glue because it vibrated the sample position. The vibration amplitudes observed by the microscope were about ±5 and ±15 µm in the vertical and horizontal directions, respectively. The vertical amplitude is about 5% of the crystal size, and almost negligible if taking the vertical spot size of 40 µm into account. However, the horizontal vibrations are not negligible and could cause unwanted irradiation of the surrounding area. In order to reduce the horizontal vibrations, we have recently devised a vibration damper, which horizontally clamps the radiation shield of the cryostat, as shown in Fig. 8 ▶. The clamping arms can be fine-tuned with micrometer feedthroughs so as not to change the sample position displayed on the microscope monitor. The ends of the arms are thermally and electrically insulated by ceramics and wired to electrical feedthroughs, which allows us to notice the mechanical contact between the arms and the grounded radiation shield. The cold stage of the cryostat is supported by the surrounding radiation shield *via* a thermal-insulating polyimide spacer located near the sample position. Hence, the vibrations of the cold stage can be damped by clamping the radiation shield. Following an off-line test, we confirmed that the horizontal vibrations were reduced to less than several micrometers when using this damper; this is negligible for 100 µm crystals. It was found that the cryostat maintained the lowest temperature when the damper clamped the radiation shield. In the case of soft X-ray ARPES, as already described, less-frequent changes of the sample angle are required than in conventional ARPES by virtue of the angle-dispersive detection covering the full Brillouin zone. Therefore, the adjustment of the clamping arms is not troublesome for our ARPES measurements. We expect that the extrinsic background signals observed in the present ARPES test will be eliminated by additional tuning of the focusing optics and use of the vibration damper for the cryostat.

## 4. Conclusions

We have developed a soft X-ray ARPES system for measuring single crystals as small as 100 µm. Using this system the band dispersions of a 120 µm × 100 µm × 80 µm Si crystal have clearly been observed at 100 K for the (111) surface cleaved *in situ*. Although some extrinsic signals coming from the area outside the crystal were observed, they will be eliminated by reducing the horizontal spot size and using a vibration damper for the cryostat. The elemental techniques described here, such as microcleaving and micropositioning, are expected to have potential capabilities for crystal sizes down to 10 µm. Spot sizes of less than 10 µm are now accessible with focusing mirrors in third-generation synchrotron facilities. By combining the above techniques with such microbeams, we expect that an ARPES system applicable to 10 µm crystals and powerful enough for investigating most small crystals of novel materials will be realised in the near future.
